# Field Metabolic Rate Is Dependent on Time-Activity Budget in Ring-Billed Gulls (*Larus delawarensis*) Breeding in an Anthropogenic Environment

**DOI:** 10.1371/journal.pone.0126964

**Published:** 2015-05-28

**Authors:** Sarah C. Marteinson, Jean-François Giroux, Jean-François Hélie, Marie-Line Gentes, Jonathan Verreault

**Affiliations:** 1 Centre de recherche en toxicologie de l’environnement (TOXEN), Département des sciences biologiques, Université du Québec à Montréal, Montréal, QC, Canada; 2 Groupe de recherche en écologie comportementale et animale (GRECA), Département des sciences biologiques, Université du Québec à Montréal, Montréal, QC, Canada; 3 Centre de recherche en géochimie et géodynamique (GEOTOP), Université du Québec à Montréal, Montréal, QC, Canada; Hokkaido University, JAPAN

## Abstract

Environmental and behavioral factors have long been assumed to affect variation in avian field metabolic rate (FMR). However, due to the difficulties in measuring continuous behavior of birds over prolonged periods of time, complete time-activity budgets have rarely been examined in relation to FMR. Our objective was to determine the effect of activity (measured by detailed time-activity budgets) and a series of extrinsic and intrinsic factors on FMR of the omnivorous ring-billed gull (*Larus delawarensis*). The experiment was conducted during the incubation period when both members of the pair alternate between attending the nest-site and leaving the colony to forage in aquatic and anthropogenic environments (city, agricultural). FMR was determined using the doubly labeled water method. Time-activity budgets were extrapolated from spatio-temporal data (2-5 days) obtained from bird-borne GPS data loggers. Gulls had low FMRs compared to those predicted by allometric equations based on recorded FMRs from several seabird species. Gulls proportioned their time mainly to nest-site attendance (71% of total tracking time), which reduced FMR/g body mass, and was the best variable explaining energy expenditure. The next best variable was the duration of foraging trips, which increased FMR/g; FMR/g was also elevated by the proportion of time spent foraging or flying (17% and 8% of tracking time respectively). Most environmental variables measured did not impact FMR/g, however, the percent of time birds were subjected to temperatures below their lower critical temperature increased FMR. Time-activity budgets varied between the sexes, and with temperature and capture date suggesting that these variables indirectly affected FMR/g. The gulls foraged preferentially in anthropogenic-related habitats, which may have contributed to their low FMR/g due to the high availability of protein- and lipid-rich foods. This study demonstrates that activities were the best predictors of FMR/g in ring-billed gulls, thus providing strong support for this long-standing theory in bioenergetics.

## Introduction

Several factors are known to modulate energy expenditure in animals including environmental variables such as, season, weather, and food availability [1,2] as well as intrinsic variables such as behavior, physiology, reproductive status and effort [1,3,4]. Considering the variability of these factors, it is not surprising that large inter-individual differences in daily energy expenditure, or field metabolic rate (FMR), have been reported in several avian species (reviewed in [5]). Determining how these variables influence energy expenditure in birds has received considerable attention in recent years. However, most studies that have attempted to relate environmental variables to FMR have been unable to establish a clear relationship [6,7]. This suggests that intrinsic elements such as activity may have a stronger impact on FMR, thus masking any effects of environmental influences [8]. Nevertheless, few studies have examined the combined influence of intrinsic and extrinsic factors on FMR in free-living animals [8].

Bioenergetics have been well studied in breeding seabirds because of their large size, colonial behavior, and relative ease of capture. A number of studies have addressed the relationships between energy expenditure and activity. However, due to the inherent challenge of measuring continuous behavior over a prolonged period of time, most of these studies have focused only on separate components of the overall activity budget. It has been shown that variables related to activity (e.g., chick provisioning rates, and proportion of time spent flying or in non-resting activities) consistently have the strongest effect on FMR compared to weather variables (within one season), age, body size or mass ([3] and references therein). Similarly, time-activity budget modeling in seabirds has been shown to best predict FMR relative to allometric equations based on body mass or thermodynamic models, thus highlighting the need for detailed information on time-allocation in FMR studies [9]. For example, energy expenditure was positively associated with the duration of foraging trips away from the nest-site [10,11], the proportion of time spent foraging [6], and the proportion of time spent flying or the number of dives [6,12] in several seabird species. In contrast, incubation appears to have low energetic implications for seabirds, sometimes being less costly than daytime resting or loafing [6], during which FMR may even become close to basal metabolic rate (BMR) [13,14]. However, complete time-activity budgets have rarely examined in relation to FMR in birds and our understanding of how high- and low-cost activities balance out is limited.

Data logging devices for monitoring bird movements are increasingly being employed to examine the effect of foraging behaviour on energy expenditure in seabirds [11,12,15]. However fine-scale time-activity budgets measured continuously over several days specifically, have yet to be examined in relation to FMR in any bird species. The ring-billed gull (*Larus delawarensis)* is a useful model to address this question because during incubation, both members of the pair share nest-site attendance [16], and all individuals alternate between bouts of low-cost incubation and higher-cost foraging activities on a daily basis. An additional limitation in our understanding of the relationship between activity-specific time-allocation and FMR in birds is that most studies have been conducted on seabirds, mainly due to the large size of tracking devices. Therefore, there is an important bias in the literature towards pelagic feeding strategies. Little information is available for omnivorous birds using terrestrial, freshwater and/or anthropogenic habitats for foraging. In such habitats, time-activity budgets may be considerably different as they provide food resources of varying quality, availability and predictability [17]. Ring-billed gulls thus additionally offer a unique opportunity to address this critical knowledge gap. Furthermore, these gulls exploit a mosaic of heterogeneous habitat types with wide inter-individual variability [18], suggesting differential energetic implications related to habitat use.

The objective of the present study was to determine the effect of activity, as measured by complete time-activity budget, as well as habitat use strategy, and selected environmental variables on FMR in ring-billed gulls breeding in an urbanized landscape. Because the effects of environmental variables are often masked by activity, we also examined how these variables related to time-activity budget to determine if they may indirectly affect FMR through behavior. We hypothesized that time-activity budget would largely explain the variation in FMR in ring-billed gulls, followed by environmental variables and foraging habitat utilization.

## Materials and Methods

Ring-billed gulls were studied during the incubation period in the spring 2011 on Deslauriers Island (45°42’45”N, 73°26’25”W) in the St. Lawrence River near Montreal (QC, Canada). This island is surrounded by a mosaic of agricultural, urban and suburban habitats, and hosts approximately 48,000 breeding pairs of ring-billed gulls [17]. During egg-laying, 200 nests containing a single egg were marked and this clutch initiation date was recorded. Once clutches were complete (three eggs), a sample of birds were randomly selected among those nests for capture throughout the entire incubation period. Birds were caught on their nest (one member per pair only) using a radio-controlled noose trap, and recaptured opportunistically 2–5 d later between the 25^th^ of April and 31^st^ of May. No injuries occurred during capture and no nests were predated while birds were being handled. Birds were sexed following euthanasia, which was part of other research objectives.

Weather data were obtained from the closest Environment Canada monitoring station (5 km) at Varennes, QC (http://www.climate.weatheroffice.gc.ca/climateData). For each bird, mean hourly temperature (°C), relative humidity (%), and wind speed (km/h) were recorded, and minimum and maximum temperatures were determined for the monitoring period. The lower critical temperature (LCT), a threshold under which heat loss exceeds energy production at rest in birds and when individuals must expend more energy to maintain a constant body temperature, was estimated using an equation for seabirds: LCT = 43.15 - 6.58 log mass - 0.26 latitude ([1], equation 11.9). The mean (± SD) LCT was 13.9 ± 0.3°C and the proportion of time (based on hourly temperatures) and the total number of hours the gulls were exposed to temperatures below this threshold were determined. The same LCT value was used for all birds because the difference between male and female LCT means was negligible (≈ 0.5°C) and would not have affected the number of hours they were exposed to lower temperatures.

### Doubly labeled water procedure

Water labeled with both heavy oxygen and hydrogen (doubly labeled water—DLW) was used to track the elimination of these two elements via respiration and thus calculate energy expenditure. The DLW solution was prepared using 35 g of water labeled as containing 97% ^18^O and 25 g of water labeled as containing 99% ^2^H (Cambridge Isotope Laboratories, MA, USA) resulting in a mixture measured to contain 60.97% ^18^O (371,000 ppm) and 37.10% ^2^H (609,700 ppm). At the initial capture, each bird was injected with 0.65 mL of DLW solution in the pectoral muscle. The needle and syringe were weighed (± 0.01 g) in the laboratory to determine the exact volume of DLW injected. The bird’s body mass was recorded, and birds were kept in a covered cage in the field to allow DLW equilibration in the body water for 1 hr [19]. An initial blood sample of 3 mL was collected from the brachial vein with a heparinized 25-gauge needle and syringe to determine enrichment at equilibrium (mean ^18^O: 3250.1 ± 14.4 ppm; ^2^H: 905.7 ± 8.8 ppm), after which the bird was released near its nesting site. Immediately following recapture 2–5 d later, birds were weighed again and 3 mL of blood was collected. Blood was centrifuged in the laboratory within 10 hrs of collection and plasma was stored in four 500 μL cryotubes at -80°C to reduce evaporative water loss until analysis.

### Isotope analysis

Plasma was thawed at room temperature and was analyzed in duplicate directly for heavy isotopes. Samples were vortexed and 200 μL were transferred onto 3 mL Labco vials for ^2^H/^1^H analysis. For ^18^O/^16^O analysis, 100 μL of plasma was diluted in 100 μL of local tap water with a known isotope ratio. The samples were analyzed on a Micromass Isoprime DI gas isotope ratio mass spectrometer (IRMS) coupled to an Aquaprep system using the equilibration method. For ^2^H/^1^H, the isotopic equilibrium required the presence of hydrophobic platinum as a catalyst [20]. After purging three times with the equilibrium gas (di-hydrogen for δ^2^H; carbon dioxide for δ^18^O), the head space was filled with 2.8 mL of gas and allowed to exchange and equilibrate with water by incubating at 40°C for 4 h to 7 h for δ^2^H and δ^18^O, respectively. A sample of the gas in the head space was directed into the IRMS, dried through a cold trap (-80°C), and analyzed in dual inlet mode against an internal reference gas. Three internal reference water samples with a range of known isotope ratios were used to correct samples for drift and normalize data to true isotope values during each analytical sequence. The internal reference waters were calibrated against Vienna Standard Mean Ocean Water (VSMOW) on the VSMOW-Standard Light Antarctic Precipitation (SLAP) scale. The δ values provided by the IRMS were converted into parts per million (ppm) by using a ^2^H/^1^H of 155.75 × 10^–6^ [21] and a ^18^O/^16^O of 2005.20 × 10^–6^ [22] for VSMOW. To determine background levels of ^18^O and ^2^H, plasma was collected from five control ring-billed gulls from this same colony, and analyzed in duplicate for O and H isotopes. Mean background levels were 1987.6 ppm for ^18^O and 147.4 ppm for ^2^H.

To validate the use of whole plasma in lieu of extracted water from whole blood, we conducted a test whereby isotope ratios obtained directly from plasma samples were compared to ratios obtained from extracted plasma water samples. Enriched plasma obtained from DLW-injected birds was pooled into 20 distinct 1 mL sample aliquots. For each pooled sample, 500 μL of plasma was lyophilized to extract the water and 10 of these paired samples were analyzed for ^2^H/^1^H ratios and ^18^O/^16^O. Isotope ratios were highly similar between the two media (linear regression: δ^2^H: R^2^ = 0.997; δ^18^O: R^2^ = 0.998; S1 Fig). Because ^2^H/^1^H ratios have previously been shown to be similar in red blood cells and plasma based on this methodology [23], removal of red blood cells from our samples was assumed to have no effect on isotope ratios.

### Calculation of field metabolic rate

The ppm values for all sets of plasma duplicates (initial and final for ^18^O and ^2^H each) were highly similar (Pearson’s correlation: 0.94 > *r*_*p*_ < 0.99), and thus one set of each was chosen at random and used for analyses rather than averaging two values which generates a midpoint rather than a mean with a calculable variation. CO_2_ production was determined using the single pool equation for FMR ([24], equation 4.17). The default dilution space ratio of 1.036 was used. The CO_2_ production was converted to FMR in kJ/d using software developed by Speakman and Lemen [25] with a respirotory quotient (RQ) of 0.85 which is a reliable assumption for birds eating a mixed diet of lipids and carbohydrates [26] as do ring-biled gulls [17]. Allometric FMR was estimated using the equation for Charadriiforms: FMR = 9.014 mass^0.655^ × [exp_10_ (latitude)]^0.0048^ ([1], equation 11.14) as an efficient mean to compare measured FMRs with the FMR that would be predicted based on previous research in several seabird species. Activity-specific FMRs for the three main activities (nest-site attendance, foraging, and flying) were extrapolated from simple linear regressions between FMR/g and the percent of time spent in a given activity using the combined males and females, and a percentage of 100.

### Time-activity budget

At first capture, a miniature GPS data logger (model GiPSy2, TechnoSmArt, Guidonia, Roma, Italy) was affixed on the bird’s two central rectrices after DLW injection. The weight of these units was 14–15 g, representing 2.5–3.7% of their body mass (mean ± standard error (SE) = 478 ± 7 g). Birds were marked with a nontoxic blue marker for recognition and at the second capture the GPS data logger was retrieved. Non-significant linear regressions between FMR and the GPS unit mass to bird body mass ratio suggested that within our sample range, smaller birds did not have increased FMR compared to larger birds by carrying the GPS unit (male: R^2^ = 0.04, *n* = 22, *p* = 0.35; female: R^2^ = 0.005, *n* = 21, *p* = 0.76).

The data loggers recorded GPS positions (± 5–10 m), speed between data points (distance between points / 4 min), date, and time every 4 min over 2–3 days. GPS positions were projected onto a high-resolution map of the area using ArcGIS (ESRI, Redlands, CA, USA). A location was assigned to each GPS position using the procedures described by Caron-Beaudoin *et al*. [18]: at the nest-site, in the colony away from the nest-site or off the colony. Off-colony positions were further defined by specific habitat types as follows: 1) “agricultural” included annual and perennial crops, 2) “urban”, included cities and suburban areas, wastewater treatment ponds, and landfills. The latter two were lumped within this category because few tracked individuals utilized these areas (*n* = 5 each) and they are associated with similar diets largely dominated by human refuse [18], and 3) “waterways” included the St. Lawrence River as well as other nearby rivers and lakes. The amount of time required for individuals to return to their nest following initial capture was extracted from the GPS trajectories.

To determine time-activity budget, each GPS position was treated like an instantaneous behavioral sample and was assigned an activity based on the location and velocity of the bird. Afterwards, all categorized GPS positions were converted into units of time by multiplying the number of data points by 4 min. The percentage of time that birds spent in different activities was determined and activities were defined as follows: 1) “Nest-site attendance” included all positions where the bird was at the nest-site including incubation; 2) “flying” included all positions where recorded speeds were above 2 km/hr; 3) “foraging” included all positions when birds were not on the colony and not flying, because lower speed activities such as walking could not be distinguished [27], and 4) “resting on the colony” included all positions where birds were on the colony, but not near the nest-site; time that was largely used for resting, preening, and loafing (S. Marteinson, pers. obs.). The total duration of time that birds engaged in particular activities (bouts) was also determined. Because birds had several short and long bouts in each activity, the following definitions were further used to isolate prolonged bouts for which mean and maximum durations were calculated; including short bouts (often as short as one data point, or 4 min) would have inhibited the accurate assessment of uninterrupted bouts: 1) “nest-site attendance bout” included periods of nest-site attendance above 1 hr as birds often came to and from the nest site a few times before settling for a long bout of nest-site attendance; 2) “travel flying bouts” included all instances where birds flew at speeds of 13 km/hr and above (determined to be a meaningful threshold by examining speed and locations in direct trajectories; and 3) “feeding trips” included all instances where birds left the colony which would encompass a series of short flying and foraging bouts. For this final measure, the total distance traveled for each feeding trip was also calculated using ArcGIS. All GPS positions where birds were located away from the colony (i.e., presumably foraging) were further categorized according to the three major habitat types listed above.

### Data analysis

Three analyses were completed using different sets of individuals to optimize sample size since FMR and complete GPS tracking were not obtained for all individuals. First, FMR/g body mass and FMR were calculated for all birds with sufficiently enriched recapture plasma (within 2–5 days), i.e. with ^2^H levels at least 10% above those of controls (only 3 individuals were thus excluded; *n* = 20 males and 23 females). Differences in metabolic rate between sexes were analysed with a t-test (FMR/g) and an ANCOVA using body mass as covariate (FMR not scaled for body mass); differences in variance between sexes were tested using a Levene’s test. Using this sample, the effect of temporal (i.e., clutch initiation date, number of days since clutch initiation, and second capture date) and environmental variables (i.e., mean wind speed, relative humidity, mean, minimum and maximum temperature, and proportion and number of h below the LCT) on FMR/g or FMR in these individuals was determined using linear regressions. For analyses using FMR, the independent variable of body mass was always included. Both metabolic rate measures (FMR and FMR/g) were analyzed for comparison; FMR/g in particular was used to express a biologically relevant measure of the amount of energy needed to power each gram of body mass and for comparison with other seabird studies (e.g., [2,6,10,11]).

Using the second data set, the time-activity budget was characterized using all individuals for which sufficient GPS tracking information was obtained as follows. Because of the variability in data recording time between capture and recapture (related to the battery duration of the devices), positions obtained during the first 40 hrs were used to standardize the data (*n* = 19 males and 18 females), and control for the proportion of hours monitored during daytime and night (included 90% of GPS-tracked birds). The proportion of time spent in the four activities over the entire 40 hrs tracking period was calculated for all birds, and for each sex separately. Activity budget variables (i.e., percent of time spent in different activities, mean bout durations, and percent of time spent foraging in different habitats) were compared between sexes using t-tests or Mann-Whitney U tests. Data were log- or square root-transformed when appropriate, though most variables were normally distributed. To determine which variables affected activity, proportional time-activity budget variables were assessed using a model selection approach. A series of linear regression models (Generalized Linear Models or GLZ) including temporal and environmental variables, sex, body mass, and foraging locations (agricultural, urban, and waterways) as predictor variables were ranked using Akaike’s Information Criterion corrected for small sample sizes (AICc). Related variables were not included in the same model (i.e., two activity measures could not be combined).

The third dataset was used to investigate the associations between time-activity budget, metabolic rate (FMR/g or FMR), and all other variables measured. Matching metabolic rate and tracking data (i.e., with no more than one hr of missing GPS positions) were obtained for 20 birds (*n* = 10 males and 10 females). Because the monitoring period varied between individuals (i.e. the entire tracking period was used to match with metabolic rate assessment), the number of hours in each activity per day was calculated and used in statistical analyses. To verify that this subset of individuals was similar to the sample formed by all individuals used in the other analyses, a series of t-tests were conducted. Metabolic rate (FMR/g and FMR) did not differ between data sets (*p* = 0.306, *p* = 0.652 respectively), nor did any activity variables (0.309 < *p* < 0.878). Linear regressions were conducted between these metabolic rate variables and activity measures combining males and females. For analyses on FMR, body mass was used as an independent variable in all models. To rank the order of importance of all variables on metabolic rate and in relation to one another, model selection was employed using a series of GLZs ranked by AICc. Because of the low sample size, only models with one predictor variable were analyzed; all activity budget, temporal, and environmental variables were assessed, each as individual model.

For all GLZs, the intercept was included. The null model for each response variable was assessed for comparison, and only models with ΔAIC_c_ values smaller, and thus more highly ranked, than the null model were considered. Models for which the parameter estimate 95% confidence intervals crossed zero were rejected. For each model, the ΔAIC_c_ was calculated as well as the weight (*w*), as a measure of its probability of being the best model to explain the variation within the set of relevant models [28]. All statistical analyses were conducted using IBM SPSS 20; when applicable, a significance level of 0.05 was employed. All means are presented with a standard error range.

### Ethics statement

Marking of ring-billed gulls was conducted under Canadian Wildlife Service banding permit 10546 while the specimens were collected under Canadian Wildlife Service scientific permit SC-23. All animal handling and field procedures were approved by the Institutional Committee on Animal Care (CIPA) of the Université du Québec à Montréal (CIPA permits nos. 646 and 768), and were in accordance with the Canadian Council on Animal Care guidelines.

## Results

### Metabolic rate and environmental variables

FMR in ring-billed gulls ranged from 229–560 kJ/d in females (mean: 377.1 kJ/d ± 20.5, *n* = 23) and 243–494 kJ/d in males (mean: 348.3 kJ/d ± 17.1, *n* = 20). FMR did not differ between sexes (ANCOVA: *F*_*1*, *43*_ = 0.042, *p* = 0.839). However, females were smaller than males (males: 511 ± 6 g; females: 433 ± 4 g; *t*_*44*_ = 8.04, *p* < 0.001) and when this difference in body mass was taken into account, the metabolic rate was significantly lower by 28% in males compared to females (FMR/g; males: 0.68 ± 0.04 kJ/g/d; females: 0.87 ± 0.05 kJ/g/d; t-test *t*_*44*_ = -3.08, *p* = 0.004). The variance did not differ between sexes for either FMR or FMR/g.

The FMR values measured in the present experiment were 1.7- and 2.0-fold lower than those calculated by the allometric equation of Ellis and Gabrielsen [1], which predicted mean FMRs of 596.6 kJ/d for females and 661.2 kJ/d for males. Mass-specific allometric mean FMRs were 5% higher in females compared to males (2.07 kJ/d/g and 1.98 kJ/d/g, respectively). In comparison to the experimental BMR for ring-billed gulls (250 kJ/d) as measured by Ellis [29], the measured FMRs were 1.4 times higher on average, and only six birds (14%) showed FMRs below this measure.

During the course of the study, hourly temperatures varied between 2.2–29.6°C (mean average: 12.7 ± 0.5°C). The total number of hours that birds were subjected to temperatures below the LCT averaged 38 ± 23 hrs (61 ± 25% of the time they were monitored). The mean average wind speed was 18.0 ± 0.7 km/hr, while the relative humidity averaged 16.2 ± 0.6%. FMR/g was positively influenced by the number of hours that birds were exposed to temperatures below the mean LCT (*R* ^*2*^ = 0.27, *p* < 0.001, *n* = 46), as was FMR (*R* ^*2*^ = 0.24, *p* = 0.004; body mass *p* = 0.673; LCT *p* = 0.001; *n* = 43). No other environmental or temporal variables were related to metabolic rate.

### Time-activity budget

All birds returned to their nests after the first capture; males returned after 9 to 213 min (mean: 44 ± 9 min), while females did so between 11 and 403 min (mean: 134 ± 34 min). Birds that did not return to their nest quickly embarked instead on typical foraging trips.

**Fig 1:**
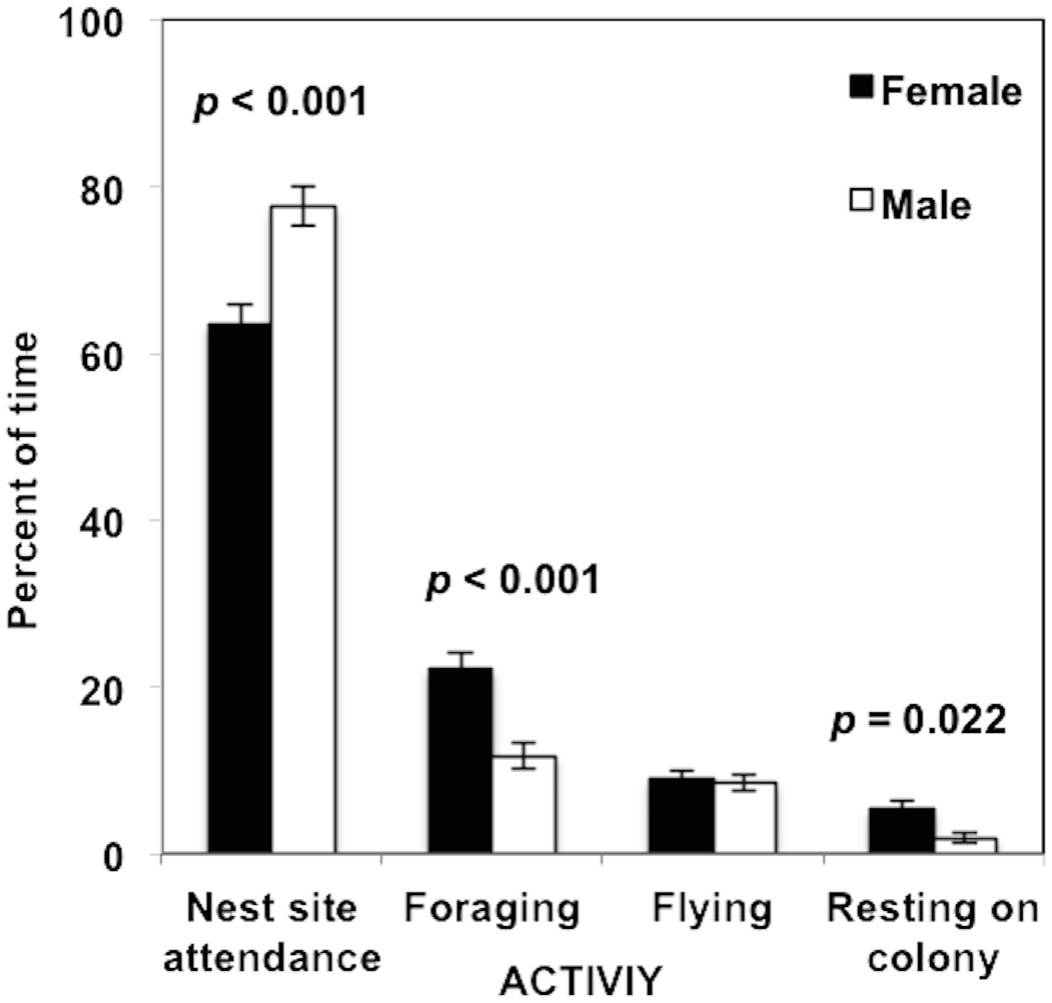
Percentage of time spent in four activities by ring-billed gulls (*Larus delawarensis*). Sample size: male *n* = 19 and female *n* = 18. Data collected near Montreal (QC, Canada) during the incubation period.

Overall, ring-billed gulls (*n* = 37) spent most of their time attending their nest-site (70.8 ± 2.0%), and males proportioned more time to this activity than females did (*t*_*35*_ = 4.30, *p* < 0.001; Fig 1). The percentage of time spent foraging followed (16.9 ± 1.5%), and females spent twice as much time as males in this activity (*t*_*35*_ = -4.33, *p* < 0.001; Fig 1). Resting on the colony was proportionally less frequent (3.6 ± 0.7%), and females spent more time in this activity than did males (*t*_*35*_ = -2.38, *p* = 0.022). Both males and females spent a similar amount of time flying (8.8 ± 0.7%) and there were no differences in mean flight speeds between the sexes. During off-colony foraging trips, females generally covered more distance each day than did males (mean of 57.0 ± 5.0 km and 48.9 ± 4.5 km in total, respectively) and tended to make longer trips (mean of 3.7 hr *vs*. 2.9 hr per trip, respectively), though these differences were not significant. Males and females exhibited similar maximum feeding trip durations (4.9 ± 0.8 h and 4.7 ± 0.6 h, respectively). Males embarked on an average of 5.5 ± 0.4 feeding trips and females on 4.5 ± 0.4 trips during the 40 hrs monitoring period. Conversely, males had on average longer travel flying bouts (males: 10.3 ± 0.9 min/bout; females: 7.9 ± 0.9 min/bout; *t*_*35*_ = 2.26, *p* = 0.030) and longer nest-site attendance bouts compared to females (males: mean of 10.7 ± 3.8 h/bout; females: 5.4 ± 0.4 h/bout; *t*_*35*_ = 2.16, *p* = 0.037). While on the foraging grounds, birds spent the majority of their time in terrestrial and anthropogenic-related habitats (agricultural: 83.4% for females and 60.9% for males; urban: 11.3% for females and 31.2% for males).

The proportion of time spent in nest-site attendance was strongly affected by sex (*w*_*i*_ = 0.79; Table 1). The proportion of time birds spent flying was affected by the date of first capture (*w*_*i*_ = 0.73), decreasing as capture date increased, followed by a positive relationship with mean temperature (*w*_*i*_ = 0.27). The proportion of time spent foraging was best explained by the model that included sex and the date of the second capture (*w*_*i*_ = 0.61; Table 1) where again, flying declined with capture date. Foraging habitats were not related to the time-activity budget.

**Table 1:** Factors affecting activities and field metabolic rate of incubating ring-billed gulls.

Response variable		Parameter estimate				
	Model	B (CI)	ΔAIC_c_	*w*_*i*_	*R* ^*2*^	Adjusted *R* ^*2*^
**% nest-site attendance**	Sex	-0.141 (-0.0204 – -0.0786)	0	0.79	0.26	0.24
	Body mass	0.001 (0.000607–0.00204)	2.64	0.21	0.26	0.24
	Null		11.13			
**% flying**	First capture d	-0.001 (-0.00126 − -0.000134)	0	0.73	0.14	0.11
	Mean temperature	0.003 (-0.000084 − -0.00706)	1.97	0.27	0.09	0.06
	Null		3.09			
**% foraging**	Sex + second capture d	0.109 (0.0647–0.153), -0.002 (-0.00463–0.00000913)	0	0.61	0.09	0.04
	Sex	0.104 (0.0584–0.150)	1.11	0.35	0.02	-0.03
	Body mass + mean temp	-0.001 (-0.00154 – -0.000487), -0.007 (-0.132–0.000462)	6.29	0.03	0.09	0.04
	Body mass	-0.001 (-0.00143 – -0.000343)	7.94	0.01	0.01	-0.02
	Null		14.60			
**FMR/g**	% nest-site attendance	0.20 (-1.561 – -0.362)	0	0.37	0.33	0.29
	Max foraging trip (h)	0.004 (0.00123–0.00581)	0.57	0.28	0.31	0.27
	Mean foraging trip (h)	0.005 (0.00102–0.00845)	2.59	0.10	0.24	0.20
	Distance traveled per d	0.004 (0.000769–0.00743)	2.96	0.09	0.23	0.18
	Max foraging trip (km)	0.005 (0.000719–0.00984)	3.44	0.07	0.09	-0.46
	% flying	2.06 (0.260–3.857)	3.53	0.06	0.20	0.16
	% foraging	0.95 (0.0275–1.862)	4.32	0.04	0.17	0.12
	Null		5.23	0.04	6.56	
**FMR**	Null		0			

Generalized linear models ranked by Akaike’s Information Criterion for small sample sizes (AIC_c_) for variables that explain the percentage of time spent in the three main activities (*n* = 37), or field metabolic rate (FMR and FMR per gram body mass in kJ/g/d) (*n* = 20) in incubating male and female ring-billed gulls (*Larus delawarensis*) near Montreal (QC, Canada). The parameter estimates (B) are shown with the 95% confidence intervals (CI) at either end. AICc = the Akaikes Information Criterion corrected for small sample sizes. ΔAIC_c_ = the difference in AIC value between a model and the top model. *w* = model weight. *R* ^*2*^ = coefficient of determination from linear regression analysis. Adjusted *R* ^*2*^ = *R* ^*2*^ adjusted to take into account several variables. LCT = lower critical temperature.

### Metabolic rate and activity

**Fig 2:**
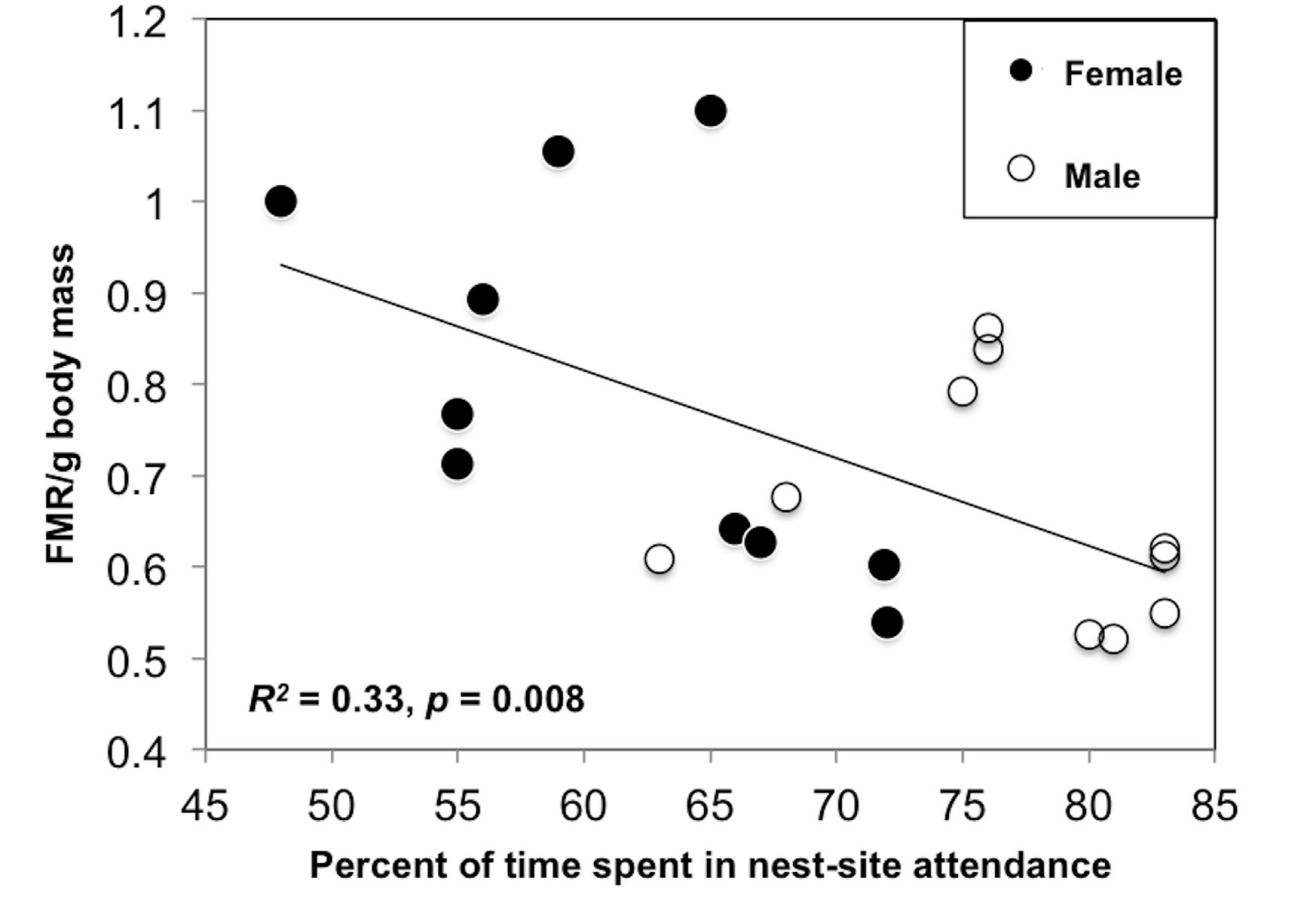
Relationships between field metabolic rate and activity in ring-billed gulls: Nest site attendance. Field metabolic rate per gram body mass was used (FMR/g). The percent of time birds spent in nest-site attendance is represented. Sample size: male *n* = 19 and female *n* = 18. Data collected near Montreal (QC, Canada) during the incubation period. Species: *Larus delawarensis*.

**Fig 3:**
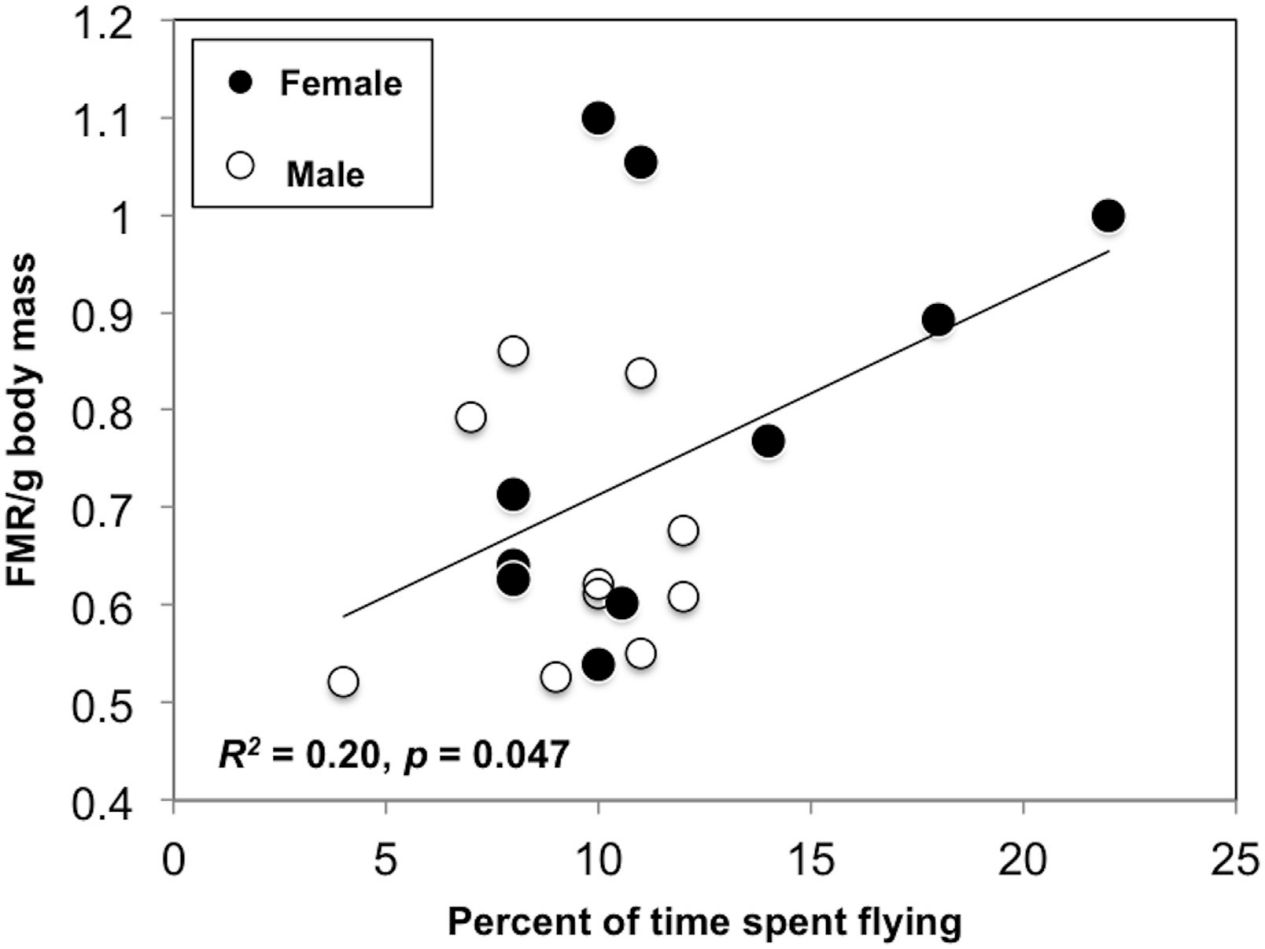
Relationships between field metabolic rate and activity in ring-billed gulls: flying. Field metabolic rate per gram body mass was used (FMR/g). The percent of time birds spent flying is represented. Sample size: male *n* = 19 and female *n* = 18. Data collected near Montreal (QC, Canada) during the incubation period. Species: *Larus delawarensis*.

**Fig 4:**
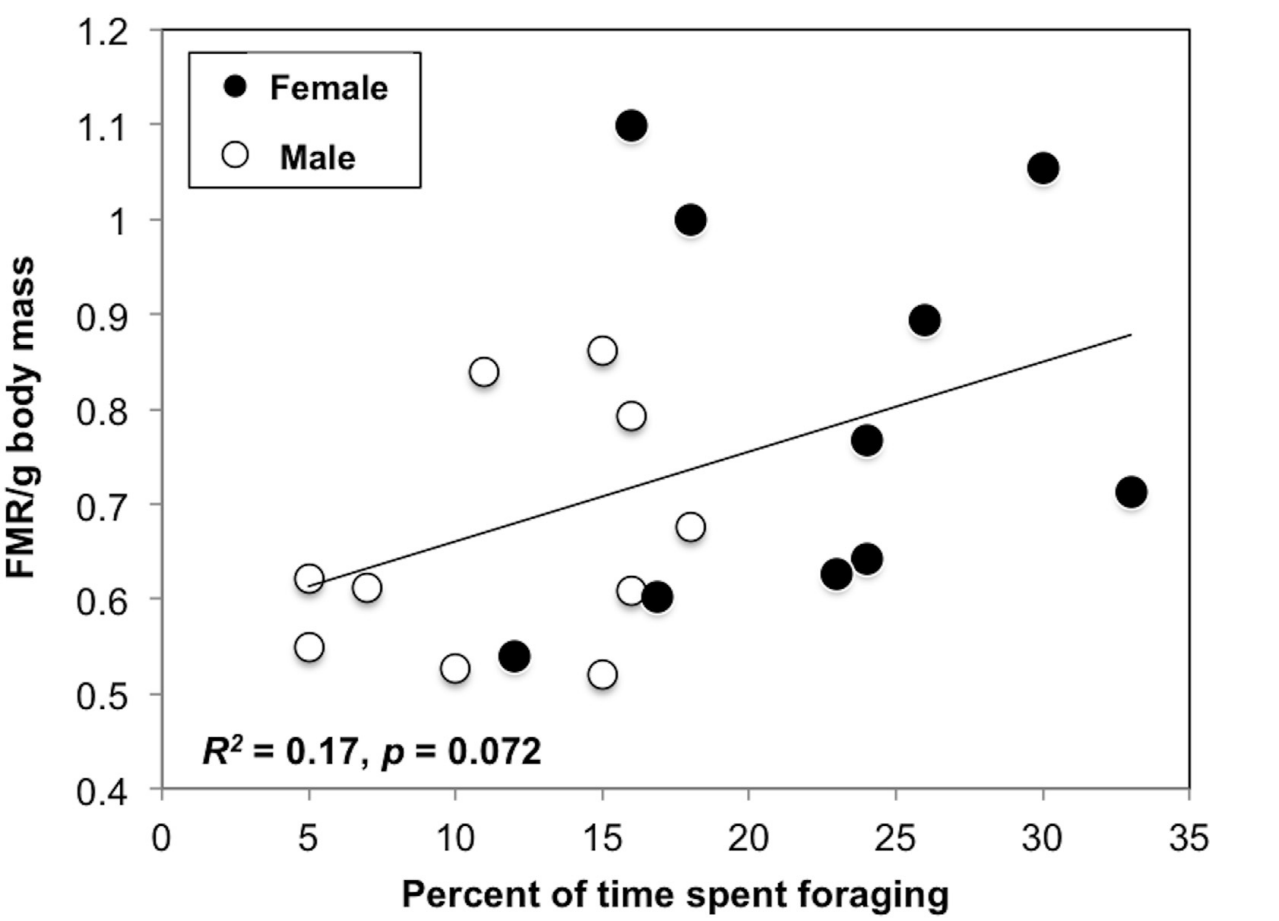
Relationships between field metabolic rate and activity in ring-billed gulls: Foraging. Field metabolic rate per gram body mass was used (FMR/g). The percent of time birds spent foraging is represented. Sample size: male *n* = 19 and female *n* = 18. Data collected near Montreal (QC, Canada) during the incubation period. Species: *Larus delawarensis*.

The most important variables affecting FMR/g (*n* = 20) were all related to the time-activity budget (Table 1). The model that best explained the variation in FMR/g was the percentage of time spent in nest-site attendance (*w*_*i*_ = 0.37); FMR/g decreased with increasing percentage of time spent in this activity (*R* ^*2*^ = 0.33, *p* = 0.008; Fig 2). The second best model was the maximum duration of foraging trips (*w*_*i*_ = 0.28), which was 75% as likely to explain the variation as the top model; FMR/g increased with increasing maximum foraging trip duration (*R* ^*2*^ = 0.31, *p* = 0.011). The third ranked model, the mean foraging trip duration, was only 27% as likely to explain the variation as the top model, thus representing a large gap in model likelihood. The subsequent five models were all variables related to foraging and flying (0.09 < *w*_*i*_ < 0.04). FMR/g increased with increasing percentage of time that the birds spent flying above 2 km/h (*R* ^*2*^ = 0.20, *p* = 0.047; Fig 3), with a similar trend observed for foraging (*R* ^*2*^ = 0.17, *p* = 0.072; Fig 4). The percentage of time spent resting on the colony and the duration of prolonged nest-site attendance or travel flying bouts were not associated with FMR/g. Foraging locations and environmental variables were not related to FMR/g in this subset of individuals for which the variation in weather conditions was more restricted due to the lower sample size. In contrast, the unscaled (body mass) measure of metabolic rate, FMR (with body mass included as an independent variable), was not affected by any activity measures. Extrapolated activity-specific FMR/g in gulls were 0.44 kJ/g/d for nest-site attendance (*R* ^*2*^ = 0.33, *p* = 0.010), 1.52 kJ/g/d for foraging (*R* ^*2*^ = 0.17, *p* = 0.076), and 2.59 kJ/g/d for flying (*R* ^*2*^ = 0.21, *p* = 0.049).

## Discussion

This study demonstrates that the time-activity budget variables are the best predictors of the inter-individual variation in FMR/g in incubating ring-billed gulls, supporting our hypothesis and the long-standing assumption in seabird bioenergetics [1,3,4]. We have demonstrated that increased time spent in lower-cost activities (e.g., nest-site attendance) reduced FMR/g, while conversely, the more time ring-billed gulls allocated to high-cost activities such as flying and foraging, the higher was their FMR/g during the incubation period. Together, time allocated to these activities explained a high proportion of variation in FMR/g where nest-site attendance accounted for 33% and maximum foraging trip duration accounted for 31%. The number of hours that birds were exposed to temperatures below their LCT also partially affected the variation in metabolic rate (27% for FMR/g and 24% for FMR).

The apparent discrepancy in the results (except for the relationship with the number of hours that birds spent below the LCT) obtained with the use of FMR/g vs. the unscaled measure to body mass (FMR) is noteworthy. FMR is usually higher in larger animals [1,4], however, in ring-billed gulls body mass was unrelated to FMR. The lack of this relationship may be due to the differences in activity between the sexes, where unexpectedly smaller birds (females) spent more time in high-cost activities. Thus the associations between metabolic rate and activity could only be extracted when metabolic rate was normalized per gram of body mass, making FMR/g a more effective measure for assessing these relationships within our data.

### Cost of incubation in seabirds

The FMR measured in ring-billed gulls was very low and was only 1.4 times higher on average than the BMR previously measured in this species [29]. This was unexpected and likely due to the considerable amount of time spent in nest-site attendance at a low projected incubating mass-specific metabolic rate of 0.44 kJ/g/d. The majority of studies that have investigated activity and FMR in seabirds have focused exclusively on the foraging period. Conversely, our study demonstrates that nest-site attendance, a low-energy activity, can be the most important factor modulating metabolic rate in birds. Ellis and Gabrielsen [1] raised the question as to whether or not sleep might have such an impact, however, the only study that examined this showed that it was not a substantial energy-saving mechanism in little penguins (*Eudiyptula minor*; [30]).

The markedly low FMR recorded in male and female ring-billed gulls adds further evidence to the growing literature demonstrating that seabirds have low energetic costs during incubation [31]. In studies examining activity-specific FMRs, other seabird species were shown to exhibit very low FMRs during incubation (FMR_inc_) compared to those measured during foraging. For black-browed albatross (*Thalassarche melanophrys*), mass-specific FMR_inc_ was 0.196 kJ/g/d, which compared to 0.611 kJ/g/d during foraging, and consistent results have been reported in four other albatross species ([14], and references therein). In addition, several studies have demonstrated that FMR_inc_ can be lower than the resting metabolic rate (reviewed in: 13) and measured BMR (reviewed in: [1,15]. This may be due to the fact that BMR is often overestimated in wild individuals due to the stress incurred by handling [15], and as a result allometric equations may concurrently overestimate BMR. An additional possible explanation is that seabirds may have reduced basal metabolic requirements during this activity. This can result from various physiological strategies in birds including changes in body composition [32] or by reduced size of energetically costly organs including kidneys and liver [33], and this is an interesting avenue for further research. It is likely that overall BMR was reduced in these birds as well since their incubating FMR was lower than the BMR measured previously for this species [29]. Notwithstanding, since ring-billed gulls spent large proportions of their time in nest-site attendance and if incubation during this activity occurred at a low metabolic rate, this would explain their low overall FMR.

### Influence of foraging on metabolic rate

Interestingly, this study shows that the duration of foraging trips and total distance traveled were more important factors affecting FMR/g than the percent of time spent foraging or flying. These findings support other studies that examined the bioenergetics of foraging trips alone, where the duration of bouts away from the nest-site increased the energetic cost of these events in a wide variety of seabirds (regardless of climate or latitude). These include black-legged kittiwakes (*Rissa trydactyla*: [10]), Leach’s storm petrels (*Oceanodroma leucorhoa*: [34]), wandering albatross (*Diomedea exulans*: [11]), northern gannets (*Morus bassanus*: [35]), and Adélie penguins (*Pygoscelis adeliae*: [36]). More recently, the total distance traveled during foraging trips has also been shown to increase energy expenditure in Cape gannets (*Morus capensis*: [12]). We can thus conclude that trip length and total distance travelled greatly influence FMR variation even when these foraging trips alternate with long bouts of nest-site attendance within the entire time-activity budget.

While our work demonstrates that the duration of feeding trips and the percentage of time spent in flight (mainly flapping) elevated FMR/g in these largely terrestrial feeders, it was not possible to pinpoint finer behavioral details related to these activities while foraging. Several studies on pelagic seabirds have been able to demonstrate which specific behaviors are the most costly for foraging birds. For example in albatrosses, individuals using highly energy-efficient gliding flight, the number of times birds landed on the water surface (i.e., number of active foraging events) was positively related to energy expenditure [11,37]. For other species that use more costly means of locomotion, time spent in flapping flight or the number of dives were positively related to FMR during feeding trips (e.g., Cape gannets: [12]). In contrast, predominantly terrestrial feeding species including ring-billed gulls, may exhibit more diverse activities of varying energetic costs over a mosaic of habitats compared to seabirds that alternate between flying over water and landing on water. Flying was the most costly activity in our study, as expected for birds using flapping flight, with a projected mass- and activity-specific metabolic rate of 2.59 kJ/g/d compared to 1.52 kJ/g/d during foraging. However, further studies are warranted to determine what specific aspects of foraging itself may be the most energetically costly for these gulls (e.g., competition with conspecifics, locomotion types, and disturbance events).

Though the duration of foraging activities elevated the energy expenditure of ring-billed gulls, the measured mass-specific FMRs (0.68–0.87 kJ/g/d) were low, being 1.7 and 2.0 times lower for females and males, respectively, compared to those predicted allometrically [1]. The FMRs in previous works used as basis to the allometric equation for Charadriiform seabirds (e.g., kittiwakes, terns, guillemots, puffins, etc.) ranged from 1.30–4.31 kJ/g/d during various breeding phases, and at latitudes ranging from 24°N to 79°N [1]. Though the low metabolic rate in the ring-billed gulls may have been best explained by the proportion of time they spent in nest-site attendance, their foraging strategies may also have had an important impact. Other Charadriiform species with similar wing-loading (e.g., kittiwakes and terns) exploit food sources at sea, and in comparison many gull species, including ring-billed gulls, are opportunistic generalists that take advantage of easily accessible human food resources. Additionally, they use largely low-energy techniques such as picking items from the ground or water surface [38]. To date, no other studies have assessed FMR in generalist Larids such as the ring-billed gull. This in itself may also explain why the Charadriiform allometric equation failed to accurately predict the FMR of ring-billed gulls, and highlights the needs for further research on how non-pelagic foraging strategies affect metabolic rate in birds.

The use of anthropogenic food resources by the ring-billed gull may have also been a factor in reducing their energy requirements. Gulls that exploit anthropogenic food resources have demonstrated large population increases locally in recent decades including the ring-billed gull breeding in the greater Montreal area (reviewed in: [39]), which also has been associated with enhanced body condition for some birds [40]. This suggests that obtaining sufficient amounts of food in such human-impacted areas may be energetically less costly [41] compared to natural food, likely due to its predictability, higher abundance and quality. Food availability has rarely been assessed in combination with FMR in birds [2] and no studies exist on birds exploiting urban and agriculturally derived food. However, when abundant resources are predictably distributed in space and time, birds can react by reducing foraging effort to save energy (e.g., [42]). Furthermore, the anthropogenic-related food items that ring-billed gulls frequently consume have high lipid and protein contents [17]. Exploiting such high-quality dietary resources may provide energy economy for these birds, both by reducing the amount of effort required to obtain necessary nutrients and by allowing them to thus allocate more time to lower-cost activities (e.g., nest-site attendance, resting, and loafing). It should be noted that not all anthropogenic food resources have a similar effect on bird bioenergetics, and availability alone cannot predict energy savings. For example, Cape gannets that rely heavily on fisheries discards extended foraging trips in length and duration (i.e., higher FMR) compared to those preying upon pelagic fish due to the lower quality of this resource [43]. Further research on urban-adapted birds using anthropogenic food resources is needed to validate this hypothesis.

### Sex and FMR

Among species, body mass generally is the strongest correlate of FMR and can be used in allometric equations [1,4]. Because ring-billed gulls are sexually dimorphic, with males being significantly larger than females, males might be expected to have higher FMR (although slightly lower mass specific FMR). However, within bird species, FMR cannot always be predicted by body mass [3], which is likely due to the low intraspecific variability in this measure [9] as well as differences in activity budgets [3]. In ring-billed gulls, the time-activity budget significantly differed between sexes, which was enough to affect their mass-specific energy expenditure. In fact, nest-site attendance, which occurred in the highest proportion, was best explained by sex alone; males spent more time in this low-cost activity than females, thus reducing their metabolic rate. Additionally, despite the fact that females were smaller, they spent more time foraging and had longer feeding trips, likely to replenish energy stores following egg-laying. This supports other studies demonstrating that the more active sex has a higher metabolic rate during reproduction [44,45], irrespective of differences in body mass, further demonstrating the needs to include measures of activity in FMR studies [9].

### Environmental factors affecting metabolic rate

The amount of time that birds were exposed to temperatures below their LCT was the only environmental variable that was related to metabolic rate (both FMR/g and FMR) when a wider range of springtime temperatures were assessed. Interestingly, this measure, which denotes the amount of time birds would have had to increase energy expenditure to thermoregulate, was more meaningful than was the mean ambient temperature, which is more traditionally used. That this measure affected FMR, even at the narrow temperature range of the spring season (during which temperatures did not fall below 0°C), suggests that it would have an even larger impact in the colder seasons of the year. Research on how it might balance out with the effect of activity during those times would be beneficial. No other environmental variables, as measured, had a direct effect on metabolic rate during this phase, however, we noted that capture date and mean temperature may have had an indirect effect on the amount of time spent flying and foraging, suggesting changes in activity as the spring and breeding period progressed. Wind in particular can be an important factor in energy expenditure for birds, and can have a positive or negative effect on FMR of seabirds depending on their mode of flight (e.g. flapping vs. gliding) [12,46, 47, 48] and direction of travel. However, despite the fact that these gulls utilize largely flapping flight to cover distance (a flight mode that can be hindered by wind), wind speed did not contribute to their energy expenditure during the incubation phase at this location. This may be because birds are also able to use winds to increase their speeds [49] and the positive and negative effects may balance out. Ring-billed gulls from this colony do not take wind direction into account when choosing foraging sites (and thus direction of flight from the colony) [27], suggesting that it may not be a limiting factor. However, a more sophisticated assessment of wind speed and direction related to that which the gulls experience during travel flight would be beneficial to further understand its effect on their FMR. Further studies on the effects of environmental and ecological variables throughout the year, across phases of the life cycle [1] and with larger sample sizes would be beneficial to further determine the importance of the environmental variables on activity and metabolic rate in birds.

## Conclusion

This study provides empirical evidence that the time-activity budget, determined continuously over several days, can be the foremost factor affecting individual variation in daily energy expenditure of free-ranging birds. By monitoring the activity and metabolic rate of ring-billed gulls, we were able to demonstrate how the percentage of time related to high- or low-cost activities, and the length and duration of foraging trips, were the strongest determinants of FMR/g. We also showed that exposure to temperatures below the LCT is important and that some factors (sex, body mass, and capture date) may have indirectly influenced metabolic rate through behaviour, helping to explain the complex interrelationships between activity, ecology, and daily energy expenditure in birds. Some variation in metabolic rate remained unexplained and further studies on the effect of other intrinsic factors including hormonal regulation and exposure to environmental contaminants is an important area of ongoing research.

## Supporting Information

S1 Fig Linear regression plots for test of isotope ratios in pooled plasma samples from ring-billed gulls (*Larus delawarensis*) injected with doubly labeled water (^2^H and ^18^O) and their matching extracted water from plasma samples. (DOCX)
