# Supplementary material for: Field Metabolic Rate Is Dependent on Time-Activity Budget in Ring-Billed Gulls (Larus delawarensis) Breeding in an Anthropogenic Environment
Source: PLoS One. 2015 May 28;10(5):e0126964. doi: 10.1371/journal.pone.0126964 (PMC4447286; doi:10.1371/journal.pone.0126964)

**Supporting Information**

**Fig. S1**: Linear regression plots for test of isotope ratios in pooled plasma samples from ring-billed gulls (*Larus delawarensis*) injected with doubly labeled water (^2^H and ^18^O) and their matching extracted water from plasma samples.


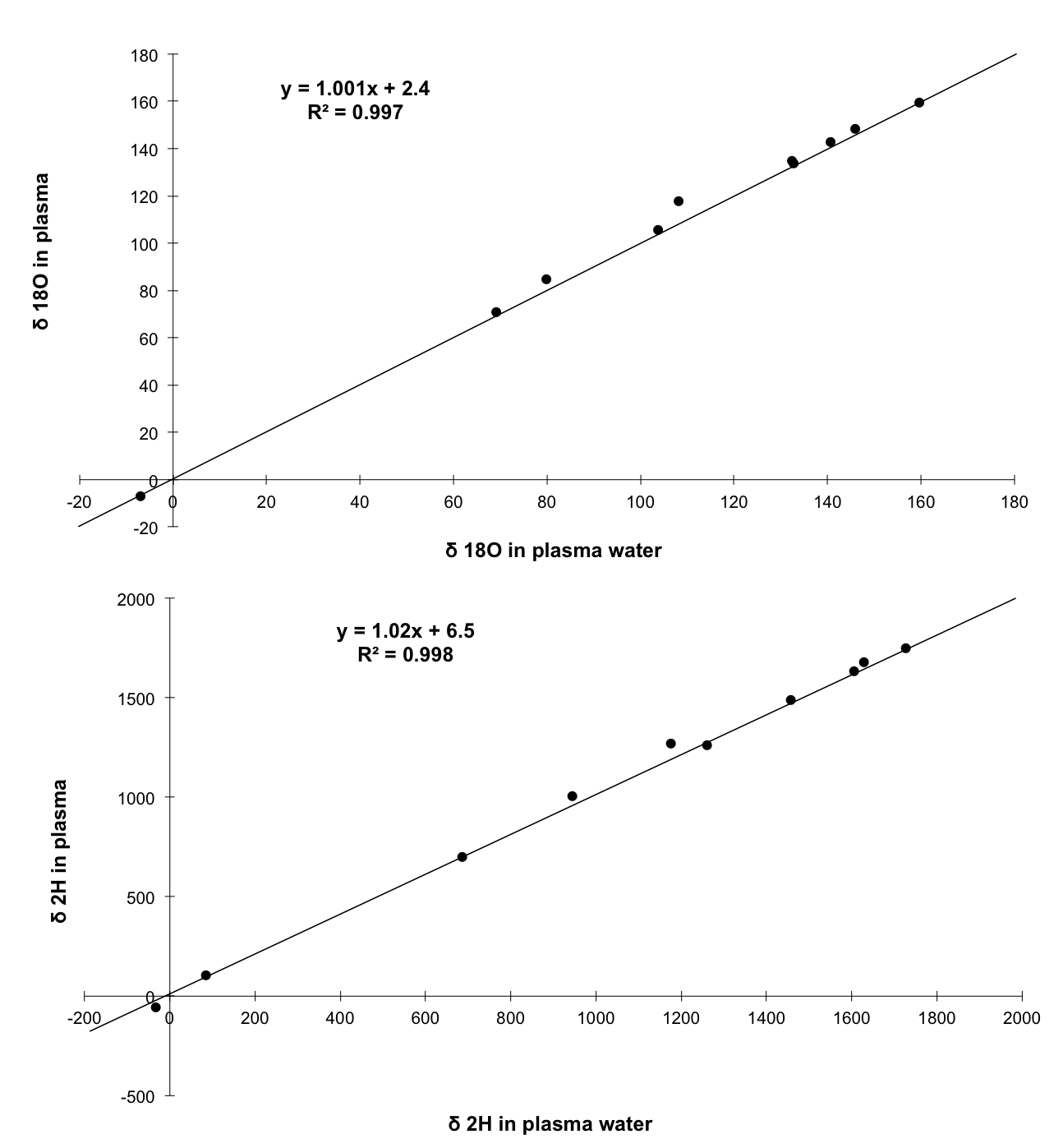

Supplement: S1 Fig — (DOCX) [file pone.0126964.s001.docx]
